# Understanding the High Prevalence of HIV and Other Sexually Transmitted Infections among Socio-Economically Vulnerable Men Who Have Sex with Men in Jamaica

**DOI:** 10.1371/journal.pone.0117686

**Published:** 2015-02-06

**Authors:** J. Peter Figueroa, Carol Jones Cooper, Jessie K. Edwards, Lovette Byfield, Shashauna Eastman, Marcia M. Hobbs, Sharon S. Weir

**Affiliations:** 1 Department of Community Health and Psychiatry, University of West Indies, Mona, Kingston 7, Jamaica; 2 Epidemiology and Research Training Unit, Kingston, Jamaica; 3 Carolina Population Center and Department of Epidemiology, University of North Carolina, Chapel Hill, United States of America; 4 Comprehensive Health Centre, Kingston, Jamaica; 5 Departments of Medicine and Microbiology & Immunology, University of North Carolina, Chapel Hill, United States of America; Public Health Agency of Barcelona, SPAIN

## Abstract

**Objectives:**

This study estimates HIV prevalence among men who have sex with men (MSM) in Jamaica and explores social determinants of HIV infection among MSM.

**Design:**

An island-wide cross-sectional survey of MSM recruited by peer referral and outreach was conducted in 2011. A structured questionnaire was administered and HIV/STI tests done. We compared three groups: MSM who accepted cash for sex within the past 3 months (MSM SW), MSM who did not accept cash for sex (MSM non-SW), and MSM with adverse life events (ever raped, jailed, homeless, victim of violence or low literacy).

**Results:**

HIV prevalence among 449 MSM was 31.4%, MSM SW 41.1%, MSM with adverse life events 38.5%, 17 transgender MSM (52.9%), and MSM non-SW without adverse events 21.0%. HIV prevalence increased with age and number of adverse life events (test for trend P < 0.001), as did STI prevalence (P = 0.03). HIV incidence was 6.7 cases/100 person-years (95% CI: 3.74, 12.19). HIV prevalence was highest among MSM reporting high-risk sex; MSM SW who had been raped (65.0%), had a STI (61.2%) and who self identified as female (55.6%). Significant risk factors for HIV infection common to all 3 subgroups were participation in both receptive and insertive anal intercourse, high-risk sex, and history of a STI. Perception of no or little risk, always using a condom, and being bisexual were protective.

**Conclusion:**

HIV prevalence was high among MSM SW and MSM with adverse life events. Given the characteristics of the sample, HIV prevalence among MSM in Jamaica is probably in the range of 20%. The study illustrates the importance of social vulnerability in driving the HIV epidemic. Programs to empower young MSM, reduce social vulnerability and other structural barriers including stigma and discrimination against MSM are critical to reduce HIV transmission.

## Introduction

Men who have sex with men (MSM) have high HIV disease burden worldwide [1]. Even in high-income countries where overall HIV epidemic trends are in decline, HIV prevalence remains high among MSM [2]. This is attributed to high per-act and per-partner HIV transmission probability of unprotected receptive anal intercourse and casual partnerships [3]. Structural issues and cultural norms such as poverty, access to services, stigma, discrimination, homophobia, gender roles and sexual identity contribute to greater vulnerability to HIV infection [4]. Social structural barriers and sexual network and partnership patterns contribute to higher HIV disease burden among black MSM in high-income countries despite black MSM reporting fewer sexual risk behaviours than other MSM [5]. Prevention efforts have had limited success in curbing HIV among MSM [6].

HIV prevalence among MSM in the Caribbean is high [3, 4] and in Jamaica was reported at 32% for nearly two decades [7, 8]. HIV infection was associated with social vulnerability, history of sexually transmitted infection (STI) and receptive anal intercourse in a 2007 survey in Jamaica [8]. However, the results cannot be generalised to MSM in Jamaica because the sample was small, of low socio-economic status and commercial sex was common. This study aimed to update estimates of HIV prevalence among MSM in Jamaica, estimate HIV incidence and explore underlying determinants of HIV infection.

## Design

An island-wide survey of MSM was conducted in Jamaica in 2011. MSM were recruited by peer referral and targeted outreach. Respondents had to be age 16 or older, self identify as MSM, complete the interview, provide blood and urine for HIV/STI tests and sign informed consent. There was no financial incentive given to participate in the study. The Ministry of Health Ethics and Medico-Legal Advisory Panel approved the study and the informed consent process. Written informed consent was obtained from participants including those aged 16 years of age which is the legal age of consent in Jamaica.

Experienced female interviewers were used including the study coordinator who was well known to the community. A structured questionnaire was administered face-to-face seeking information on socio-demographic status, HIV awareness, risk perception, sexual behaviour, condom use, STI symptoms, health seeking behaviour, drug use and social vulnerability. Sensitive questions were answered by the participant privately on an answer sheet, which he placed in an envelope. Respondents unable to complete the answer sheet were assessed as having low literacy. The interviewer assessed socio-economic status using a scale of 1 (low) to 10 (high). Interviews took place privately at mutually agreed sites including private homes, selected clinics and medical offices, and in the interviewer’s car. Blood and urine samples were taken and transported to the laboratory.

HIV testing was done using the Determine rapid test. Confirmation of HIV positive tests was done at the National Laboratory using an ELISA algorithm and Western blot. Syphilis testing was done using Bioline with confirmation of positive results using TPPA. A positive syphilis test was interpreted as “ever infected”. MSM with a positive TPPA and a TRUST titre of 8 or higher were considered to be currently infected. First catch urine samples were collected in a urine cup and 2mls of urine was transferred to a Gen-Probe tube within 24 hours of collection. Gen-Probe tubes were stored in the refrigerator at 5°C until testing. Gen-Probe ATIMA Combo2 was used to test for *Neisseria gonorrhoea* and *Chlamydia trachomatis*. Initial positive and indeterminate tests were repeated. Confidential results were given to participants free of cost, with counseling, treatment and referral as indicated. We use the term “other STI” to mean any STI other than HIV.

Data were double entered and verified. Frequencies, cross-tabulations by HIV status, prevalence odds ratios and 95% Confidence Intervals (CI) were prepared using SAS (SAS Inc. Cary, NC) adjusting for clustering of the sample by city. We used the proximate-determinants model as the conceptual framework [9]. We compared the characteristics and risk factors for HIV infection between MSM who accepted cash for sex in the past 3 months (MSM sex workers – MSM-SW) and MSM not accepting cash for sex (MSM non-SW). We also compared risk factors for HIV infection between MSM with adverse life events (ever raped, jailed, homeless, victim of violence or low literacy) and MSM without adverse life events (including both SW and non-SW). These variables are considered to be markers of social vulnerability and each of them was significantly associated with HIV infection on univariate analysis of the sample of MSM. We estimated HIV prevalence by MSM subgroup and a crude HIV incidence based on 49 MSM who tested negative in the 2007 survey and were retested in 2011.

We used binomial regression to estimate crude and age-adjusted prevalence ratios comparing the prevalence of HIV and other STIs between MSM with and without selected social determinants of health. In all models, age was modeled as a continuous variable using a restricted quadratic spline, which is a piecewise polynomial function that allows flexibility in the functional form of relationship between the continuous predictor and the outcome. Use of a spline allowed us to model the association between age and the outcome flexibly instead of assuming a linear increase in prevalence of the outcomes for each unit increase in age [10].

## Results

A total of 449 MSM completed interviews and blood tests. The refusal rate was 4.2%. Participants were enrolled through behaviour change workshops (145 or 32.3%), verbal referral by participants (109 or 24.3%) the study coordinator’s MSM contacts (77 or 17.1%), use of referral cards from participants (46 or 10.2%), interviewers at social events and parks (43 or 9.6%) and through a NGO service point (29 or 6.5%). Subject characteristics were generally similar among the MSM according to recruitment method.

Two-fifths (40.8%) of respondents lived in Kingston and St Andrew and 22.1% in Montego Bay. Most (75.0%) MSM described their gender as male, 21.2% as female and 3.8% as transgender (Table 1). The majority were bisexual (56.8%), 16–24 years of age (61.7%), of low socio-economic status (ranked 1–4 on a scale of 10) (72.4%), unemployed (57.0%), and had not completed secondary school (73.5%). Most (77.5%) were not comfortable telling others that they were MSM. The majority (57.2%) had either been raped, jailed, homeless, a victim of violence and/or low literacy while 95 (21.2%) reported accepting cash for sex in the past 3 months. Transactional sex, defined as receiving or giving gifts or goods for sex but not cash, was reported by 161 or 35.9% of MSM. Nearly one third had met a new sex partner on the street (29.2%) or on the Internet (30.7%). Most (78.8%) MSM ever had an HIV test. A minority (15.4%) knew the HIV status of their main male partner and had disclosed their own status.

**Table 1:** Characteristics of 449 men who have sex with men in Jamaica by engagement in sex work 2011.

	MSM accepting cash for sex in past 3 months		MSM not accepting cash for sex		P-value		Total
	N	%	n	%		n	%
All	95	21.2	354	78.8		449	100
Demographics							
Age: 16–24 years	64	67.4	213	60.2	0.2	277	61.7
25+	31	32.6	141	39.8	0.2	172	38.3
Self-identified as Female	29	30.5	66	18.6	0.03	95	21.2
Bisexual	59	62.1	196	55.5	0.3	255	56.8
Employed (full or part-time)	26	27.4	167	47.2	0.001	193	43.0
Currently married	13	13.7	23	6.5	0.02	36	8.0
Social Vulnerabilities							
Interviewer assesses participant to have very low SES (1–2 on scale 1–10)	58	61.1	96	27.1	0.00001	154	34.3
Low literacy	15	15.8	38	10.7	0.17	53	11.8
Low comfort telling others he is MSM	72	75.8	276	78.0	0.4	348	77.5
Type of Adverse Life Events							
Has experienced physical violence	37	38.9	89	25.1	0.01	126	28.1
Ever been to jail	37	38.9	82	23.2	0.002	119	26.5
Ever spent night outside	36	37.9	47	13.3	0.00001	83	18.5
Ever been raped	18	18.9	54	15.3	0.4	72	16.0
Disinhibiting Factors							
Perceived risk of becoming HIV +ve							
No or little chance of getting HIV	54	56.8	204	57.6	0.9	258	57.5
Met new sexual partner on street	44	46.3	87	24.6	0.0001	131	29.2
Met new sexual partner on internet	37	38.9	101	28.5	0.053	138	30.7
Protective Factors							
Ever tested for HIV	71	74.7	283	79.9	0.3	354	78.8
Tested in the past 12 months	52	54.7	208	58.8	0.47	260	57.9
Seen this risk card	76	80.0	267	75.4	0.3	343	76.4
Carrying condom seen by interviewer	28	29.5	67	18.9	0.026	95	21.2
Knowledge of HIV transmission	55	57.9	209	59.0	0.8	264	58.8
Knows HIV status of main male partner and has disclosed own status	9	9.5	60	16.9	0.02	69	15.4

Two thirds (64.6%) said they had receptive anal intercourse; 42.8% said they had both receptive and insertive anal intercourse (Table 2). A minority reported having 5+ one-night stands (15.6%) or 5+ new male partners in the past 12 months (14.7%). Among MSM with a main partner (60.0%) a majority (57.4%) reported that they thought that their partner had other sex partners. A majority of MSM reported using a condom at last sex; however, only 12.0% reported always using a condom. Half (49.2%) of the respondents had visited a health clinic in the past 12 months; of these 86.5% were satisfied with the care received. Two thirds (76.4%) of the sample had seen the risk card used by the HIV program outreach educators. One third (33.9%) said a doctor told them they had an STI.

**Table 2:** Proximate determinants of increased exposure to HIV among 449 men who have sex with men in Jamaica by engagement in sex work 2011.

	MSM accepting cash for sex in past 3 months		MSM not accepting cash for sex		P—value	Total	
	n	%	n	%		n	%
All	95	21.2	354	78.8		449	100
Proximate Determinants of Increased Exposure to HIV							
Receptive anal intercourse	59	62.1	231	65.3	0.5	290	64.6
Insertive & receptive anal intercourse	44	46.3	148	41.8	0.4	192	42.8
5 or more one night stands	27	28.4	43	12.2	0.0001	70	15.6
5 or more new male partners in past 12 months	29	30.5	37	10.5	0.0001	66	14.7
2 or more partners in 72 hours	32	33.7	60	16.9	0.001	92	20.5
Ever told by a doctor that he has STI	36	37.9	116	32.8	0.3	152	33.9
Proximate determinants of Transmission Given Exposure							
Always uses a condom	12	12.6	42	11.9	0.8	54	12.0
Does not always use a condom	83	87.4	312	88.1	0.8	395	88.0
Condom at last sex (top)	61	64.2	199	56.2	0.7	260	57.9
Condom at last sex (bottom)	53	55.8	188	53.1	0.7	241	53.7
Infection with any STI including HIV	46	48.4	135	38.1	0.07	181	40.3
Infection with any STI other than HIV	17	17.9	52	14.7	0.4	69	15.4
Chlamydia	9	9.5	31	8.8	0.8	40	8.9
Gonorrhea	3	3.2	5	1.4	0.3	8	1.8
Syphilis (Bioline and TPPA positive)	3	3.2	10	2.8	0.9	13	2.9
HIV Status							
Infected with HIV	39	41.1	102	28.8	0.02	141	31.4

The characteristics of the MSM-SW and MSM non-SW were generally similar. However, MSM-SW were significantly more likely to self-identify as a female, be unemployed and of very low socio-economic status, have a higher number of high risk sex partners and to have been homeless, jailed or suffer violence.

HIV prevalence among the sample of 449 MSM was 31.4%. HIV prevalence was significantly higher among the 95 MSM SW (41.1%), 257 MSM with adverse life events (38.5%) and 17 transgender MSM (52.9%) (Table 3). Among 167 MSM who did not exchange cash for sex and had no adverse life event, HIV prevalence was 21.0%. HIV prevalence was similar among MSM reporting transactional sex (28.6%) and those reporting neither cash nor gifts for sex (27.6%).

**Table 3:** HIV Prevalence among MSM Sub-Groups in Jamaica in 2011: MSM sex workers, MSM non-sex workers and MSM with and without adverse life events.

MSM Sub-group	N	% HIV Prevalence
All MSM in survey	449	31.4
**MSM sex workers** (accept cash for sex In past 3 months)	95	41.1
MSM SW with adverse life events	70	45.7
MSM SW without adverse life events	25	28.8
**MSM non sex workers** (MSM not accepting money for sex)	354	28.8
MSM non SW with adverse life events	187	35.8
MSM non SW without adverse life events	167	21.0
All MSM with adverse life events	257	38.5
All MSM without adverse life events	192	21.9

HIV prevalence increased significantly with age (from 24.2% among 16–24 years to 43.0% among those 25+ years). Among all MSM, HIV prevalence was highest among MSM reporting 5+ one night stands (55.7%), 5+ new sex partners in past 12 months (51.5%) and those told by a doctor they had a STI (52.0%) or who self identified as female (45.8%). For nearly all variables, HIV prevalence was higher among MSM SW and MSM with adverse life events compared with other MSM (no cash-for-sex, no adverse life event). HIV prevalence was highest for MSM SW who had been raped (65.0%), those reporting 5+ one night stands (67.6%) or 5+ new sex partners in the past 12 months (56.8%), those a doctor told they had STI (61.2%) or who self identified as female (55.6%). HIV prevalence was relatively lower among MSM who always used a condom (14.8%), said they had no or little chance of getting HIV (15.9%) or were bisexual (21.2%). Prevalence of other STI was highest among MSM who were currently married (36.1%) and those with a main female partner (22.6%).

**Fig 1:**
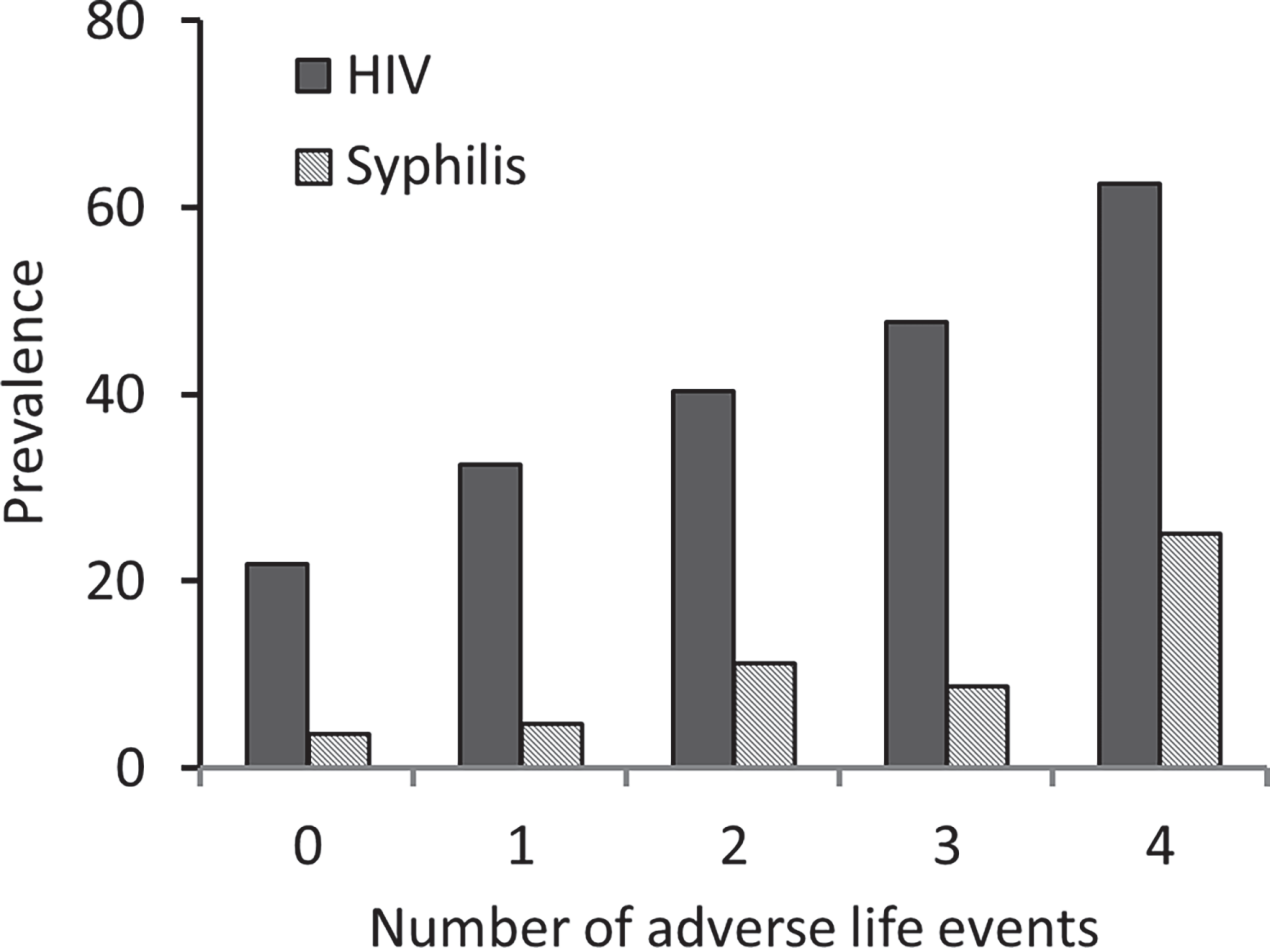
HIV and Syphilis Prevalence among MSM by Number of Adverse Life Events 2011.

HIV prevalence increased significantly with number of adverse life events (P < 0.001) (Fig. 1), as did STI prevalence (P = 0.03) (data not shown). There were 49 MSM who participated in the 2007 survey, who were HIV negative at the time and who were retested in this survey in 2011 with 11 of them found to be HIV positive. HIV incidence was estimated at 6.7 cases/100 person-years (95% CI: 3.74, 12.19).

Risk factors for HIV infection were analysed for three MSM subgroups: MSM SW, MSM non-SW, and MSM with adverse events (including both SW and non-SW). Significant risk factors common to all three subgroups were participation in both receptive and insertive anal intercourse, high-risk sex, and being told by a doctor that they had a STI (Tables 4 and 5). There were no other significant risk factors for HIV infection among MSM SW. However, the number of MSM SW (95) was relatively low. Age and very low socio-economic status were significant risk factors for HIV infection among MSM non-SW and MSM with adverse life events. Other significant risk factors among MSM non-SW were self-identifying as a female, and ever jailed, raped, or doing an HIV test. Among MSM with adverse events other significant risk factors were having a condom at the time of interview, knowledge of HIV transmission, not always using a condom and testing positive for a current STI.

**Table 4:** Selected Risk Factors for HIV infection among 354 MSM non-Sex Workers in Jamaica 2011.

Risk Factor	HIV+ve /Total	% HIV Prevalence	Odds Ratio	95% Confidence Interval
Age: 16–24 years	44/213	20.7	1	
25 + years	58/141	41.1	2.7	1.19–6.03
Self-identified as Female	27/66	40.9	2.0	1.00–3.86
Bisexual	36/196	18.4	0.3	0.17–0.67
Interviewer assesses participant to have very low SES (1–2 on scale 1–10)	42/96	43.8	2.6	1.22–5.39
Low literacy	17/38	44.7	2.2	0.93–5.22
Ever been to jail	33/82	46.3	2.0	1.00–3.92
Ever spent night outside	16/47	34.0	1.3	0.68–2.57
Ever been raped	18/54	33.3	1.3	1.09–1.66
Perceived risk of becoming HIV +veNo or little chance of getting HIV	28/204	13.7	0.2	0.09–0.29
Ever tested for HIV	91/283	32.3	2.6	1.03–6.46
Insertive & receptive anal intercourse	51/148	34.5	1.6	0.97–2.62
5 or more one night stands	21/43	48.8	2.7	1.08–6.82
5 or more new male partners in past 12 months	19/37	51.4	3.0	1.59–5.56
Ever told by a doctor that he has STI	57/116	49.1	4.1	2.24–7.67

**Table 5:** Selected Risk Factors for HIV infection among 257 MSM with Adverse Life Events in Jamaica 2011.

Risk Factor	HIV+ve /Total	% HIV Prevalence	Odds Ratio	95% Confidence Interval
Age 25 + years	61/118	51.7	2.8	1.65–4.90
Very low SES (1–2 on scale 1–10)	53/113	46.9	1.9	1.08–3.29
Perceived risk of becoming HIV +veNo or little chance of getting HIV	28/135	20.7	0.2	0.10–0.36
Met new sexual partner on street	42/88	47.7	1.8	0.93–3.46
Carrying condom seen by interviewer	36/64	56.3	2.7	1.06–6.67
Knowledge of HIV transmission	61/141	45.4	1.6	1.10–2.22
Knows HIV status of main male partner and has disclosed own status	13/38	34.2	0.7	0.46–1.05
Receptive anal intercourse	70/158	44.3	1.9	0.92–3.89
Insertive & receptive anal intercourse	51/105	48.6	2.0	1.31–3.21
5 or more one night stands	27/46	58.7	2.7	1.80–4.17
5 or more new male partners in past 12 months	23/44	52.3	2.0	1.04–3.76
2 or more partners in 72 hours	32/64	50.0	1.9	0.96–3.70
Accept cash for sex in past 3 months	32/70	45.7	1.5	0.84–2.71
Ever told by a doctor that he has STI	62/98	63.3	5.7	1.85–17.39
Always uses a condom	6/27	22.2	0.4	0.19–0.94
Does not always use a condom	93/230	40.4	2.4	1.06–5.32
Condom at last sex (bottom)	52/138	37.7	0.6	0.53–0.74
Infection with any STI other than HIV	24/46	52.2	2.0	1.23–3.17

Having a perception of being at no or little risk of HIV infection was significantly inversely associated with HIV infection in all three subgroups. Being bisexual was protective among MSM non-SW while always using a condom, using a condom at last receptive anal intercourse and disclosure of HIV status were all protective among MSM with adverse life events. Age adjusted prevalence ratios for selected underlying determinants of HIV are given in Table 6.

**Table 6:** Crude and Age Adjusted Prevalence Ratios of Selected Underlying Social determinants of HIV among MSM in Jamaica 2011.

Characteristic	Crude PR	Crude 95% CI	Adj PR	Adj 95% CI
Cash for sex (accepts or pays)	1.51	1.14, 1.99	1.41	1.07–1.85
Cash for sex (accepts only)	1.42	1.06, 1.91	1.50	1.13–1.98
Self-identifies as female	1.67	1.27, 2.20	1.94	1.52–2.48
Interviewer identified as very low SES (1 or 2 on scale of 1–10)	1.84	1.41, 2.40	2.26	1.77–2.87
Low literacy	1.61	1.17, 2.23	1.45	1.06–1.98
Ever slept outside/ homeless	1.48	1.10, 2.00	1.35	1.01–1.81
Ever been in jail	1.52	1.16, 2.00	1.36	1.04–1.77
Experienced personal violence	1.54	1.17, 2.04	1.53	1.17–2.00
Ever raped	1.39	1.00, 1.92	1.34	0.99–1.83
Number of adverse life events				
0	1		1	
1	1.50	1.03, 2.14	1.30	0.93–1.83
2	2.22	1.07, 4.60	1.70	0.86–3.34
3	3.30	1.10, 9.85	2.21	0.80–6.11
4	4.91	1.14, 21.12	2.88	0.74–11.17
5	7.30	1.19, 45.28	3.75	0.69–20.42

PR: prevalence ratio; CI: confidence interval; Adj: adjusted

Adjusted prevalence ratios are adjusted for age using a restricted quadratic spline

## Discussion

The HIV prevalence of 31.4% among MSM in this survey was high and similar to previous surveys [7, 8]. However, the results cannot be generalised to MSM in Jamaica because the survey was not representative. Many participants were MSM sex workers, of low socio-economic status and/or socially vulnerable. HIV prevalence was very high among MSM SW (41.1%), those with adverse life events or low literacy (38.5%), and transgender MSM (52.9%). In contrast, HIV prevalence was 21.0% among MSM non-SW who had no adverse life events. Based on the findings and characteristics of the sample, we assess that HIV prevalence among MSM in Jamaica may be in the range of 20%, but unlikely to be lower than 15%.

We thought it important to analyse MSM sex workers separately from other MSM because there were significant differences in their characteristics especially with respect to risk factors previously shown to be associated with HIV infection and markers of social vulnerability. The distinction between MSM and MSM sex workers was not made in describing the global epidemiology of HIV among MSM [1, 3, 4] or in the other excellent papers in the Lancet series [11]. We suggest that more attention be paid to cash for sex among MSM in future studies because this may explain some of the variability in HIV prevalence in different surveys. Previous surveys in Jamaica have included MSM sex workers, which has inflated the HIV prevalence reported [7].

Transactional sex, defined as receiving or giving gifts or goods for sex but not cash, was common among MSM. However, HIV prevalence among MSM practicing transactional sex was not significantly higher than that found among MSM non-SW, so these subgroups were combined in our analysis. Transactional sex among heterosexuals in Jamaica is thought to increase risk of HIV [7]. Transactional sex may not have appeared as a risk factor among MSM because there were other more important determinants of HIV infection such as receptive anal intercourse, multiple sex partners, casual sex and other STI as well as greater likelihood of exposure to an HIV positive partner within their sexual networks.

A significant finding was the impact of adverse life events and low literacy on HIV prevalence, suggesting that social vulnerability is a critical factor contributing to high HIV prevalence among MSM in Jamaica and elsewhere. We used the proximate-determinants model as the conceptual framework for this survey [9]. Markers of social vulnerability such as adverse life events and low literacy are considered to be significant underlying determinants of HIV infection. HIV prevalence was highest (65.0%) among MSM sex workers who were ever raped, suggesting that the rape infected them or their self-esteem was damaged thus reducing their ability to practice safe sex. Established risk factors or proximate determinants of HIV infection such as receptive anal intercourse and casual sex partners were common among MSM with underlying determinants of HIV as well as significantly associated with HIV infection which is consistent with what one would expect.

HIV prevalence among MSM in Jamaica was high relative to most surveys of black MSM across the African Diaspora [4]. Higher HIV prevalence was found among black MSM in recent surveys in Cape Town and Johannesburg, South Africa; Kalindi and Mombasa, Kenya [12]; as well as Atlanta and Baltimore, USA. Some of these studies include MSM sex workers. Other cities such as Dakar, Senegal; Durban, South Africa and Florida, USA had HIV prevalence among MSM similar to that among non-sex worker MSM in Jamaica [4].

The HIV incidence among these MSM was very high (6.7/100 person years) indicating that HIV continues to spread rapidly among socially vulnerable MSM in Jamaica despite concerted prevention efforts. In Mombasa, Kenya HIV incidence among MSM sex workers was estimated at 8.6 (CI 5.7–12.7) per 100 person years and among homosexual men 3.6 (CI 1.4–9.8) [13]. In the USA the overall HIV incidence was estimated to have been stable between 2006 and 2009 but to have increased by 48% among young black MSM aged 13–29 years [14].

The HIV epidemic among MSM in Jamaica and the Caribbean does not readily fit into the framework proposed by Beyrer et al. for characterising HIV epidemics in MSM in wider epidemiological contexts [1, 3]. He described four scenarios for low and middle-income countries: 1. MSM predominance, 2. Injection drug use, 3. Widespread epidemics in heterosexual people and 4. Contexts where heterosexual spread, sex work, MSM risks, and injection drug use were all contributors to HIV spread [3]. None of the four scenarios described apply to either Jamaica or the Caribbean where the HIV epidemic is generalised at a population prevalence of 1.7% and 1% respectively [15], where heterosexual transmission is the primary mode of spread [7, 16], and HIV prevalence is high among MSM but they do not form the predominant contribution to HIV in the population as in South America [17].

The only variables significantly associated with reduced HIV infection were low perceived risk, using a condom always (among MSM with adverse events) and bisexuality (among MSM non-SW). Only 12.2% of MSM said that they always used condoms and some of them may have known that they were HIV positive prior to the survey. Reported condom use at last sex was relatively high but social desirability bias likely influenced reported condom use. Given the high per-act probability of HIV transmission with anal sex, anything less than consistent condom use is unlikely to be protective, especially in the presence of other STI. It is not clear why there was an association between bisexuality and reduced HIV infection among MSM non-SW.

Current STI other than HIV were found in 15.4% of MSM, which is likely an underestimate because specimens were urine-based and not rectal. This is a limitation of the survey and weakens the analysis using STI other than HIV as an outcome. Despite this shortcoming, many (42.0%) MSM and 61.5% of MSM sex workers with a current STI were HIV infected reflecting the strong synergistic relationship between HIV and other STI [18]. A history of infection with another STI was also strongly associated with HIV infection, as in 2007 [8].

Access to services appeared high. The proportion of MSM doing an HIV test in the past 12 months increased from 30.8% in 2007 [8] to 57.9% in 2011. The majority (76.1%) had seen the risk card used by outreach educators, suggesting that prevention services were reaching the most vulnerable MSM. The impact of these prevention services in slowing HIV spread cannot be assessed; however, they appear to have limited effect. These MSM appeared to assess their risk fairly accurately. Nevertheless, they may overestimate their ability to avoid HIV infection or many of them may be fatalistic towards getting HIV. Another challenge is the failure of most of these MSM to disclose their HIV status to their sex partners or to use condoms consistently even when they are aware they are HIV positive.

Half (49.2%) of the MSM had visited a health centre in the past 12 months and most (86.5%) of them were satisfied with the service. The level of satisfaction reported is comparable to that found in surveys of persons attending health facilities in Jamaica and runs counter to the perception that MSM are not treated well by health staff. We do not know why half of the MSM did not use the public health services. However, in a society that strongly disapproves of homosexuality and where stigma associated with MSM and HIV is strong, hesitation among MSM in seeking services even where they are readily available is understandable. Most MSM surveyed (78%) were not comfortable revealing their sexual orientation to others and many failed to attend the physician for free STI treatment despite numerous reminders. The strong stigma also likely contributes to self-stigma and increased risk taking [19–21].

There were limitations in this survey. The sample was not representative of all MSM in Jamaica. Recruitment may have biased the results towards MSM who were more likely to be in touch with the HIV program. However, MSM who were socially vulnerable, of lower socio-economic status and MSM sex workers were disproportionately recruited, suggesting that the program is targeting the MSM most in need. If this were the case, the crude HIV prevalence would overestimate the true HIV prevalence among MSM in Jamaica. Another limitation was the failure to obtain rectal swabs, so STI prevalence is underestimated. Some social desirability bias may be present for certain questions such as condom use.

In conclusion, HIV prevalence was very high among MSM sex workers (41.1%) in Jamaica, high among non-sex worker MSM (28.8%), and very high among MSM with adverse life events (38.5%). On the other hand HIV prevalence among MSM non-SW and without adverse events was 21.0%. Based on the characteristics of the sample, including the low socio-economic status and high social vulnerability, we estimate that the HIV prevalence among MSM in Jamaica is probably in the range of 20%. The high HIV prevalence among MSM with adverse life events emphasizes the critical role of social vulnerability in contributing to the HIV epidemic. HIV prevention services appear to be accessible but of limited effectiveness due to several factors including high social vulnerability, commercial sex, high number of casual sex partners, other STI, and failure to use condoms consistently. Prevention efforts in Jamaica need to be reviewed in the context of these findings and the prevention options currently available in order to achieve an appropriate combination of approaches with wide coverage and greater effectiveness. Programs to empower young MSM and reduce social vulnerability and other structural barriers including stigma and discrimination against MSM are critical to reduce HIV transmission.
